# OSIEshort: A small stimulus set can reliably estimate individual differences in semantic salience

**DOI:** 10.1167/jov.20.9.13

**Published:** 2020-09-18

**Authors:** Marcel Linka, Benjamin de Haas

**Affiliations:** Experimental Psychology, Justus Liebig Universität, Giessen, Germany; Experimental Psychology, Justus Liebig Universität, Giessen, Germany

**Keywords:** eye movements, individual differences, free-viewing, saliency

## Abstract

Recent findings revealed consistent individual differences in fixation tendencies among observers free-viewing complex scenes. The present study aimed at (1) replicating these differences, and (2) testing whether they can be estimated using a shorter test. In total, 103 participants completed two eye-tracking sessions. The first session was a direct replication of the original study, but the second session used a smaller subset of images, optimized to capture individual differences efficiently. The first session replicated the large and consistent individual differences along five semantic dimensions observed in the original study. The second session showed that these differences can be estimated using about 40 to 100 images (depending on the tested dimension). Additional analyses revealed that only the first 2 seconds of viewing duration seem to be informative regarding these differences. Taken together, our findings suggest that reliable individual differences in semantic salience can be estimated with a test totaling less than 2 minutes of viewing duration.

## Introduction

In order to make sense of our visual environment, we constantly move our eyes. This allows us to observe fixed and moving objects with high foveal resolution and to inspect regions of interest while ignoring many others (Gegenfurtner, 2016). To explain this selection process, models of attentional guidance have tried to predict gaze behavior based on, for example, task relevance (Fecteau & Munoz, 2006); low-level image features, such as local contrast for color, intensity, and orientation (Harel, Koch, & Perona, 2006; Itti & Koch, 2000); and high-level, semantic features, such as faces or text (Xu, Jiang, Wang, Kankanhalli, & Zhao, 2014). What these models all have in common is that they try to predict a typical observer and treat individual differences as noise.

More recent findings, however, show stable, trait-like differences in eye movements; for example, oculomotor measures such as mean saccade amplitude, smooth pursuit duration, or mean fixation duration vary reliably among people (Bargary, Bosten, Goodbourn, Lawrance-Owen, Hogg, & Mollon, 2017; Castelhano & Henderson, 2008; Henderson & Luke, 2014). Moreover, recent twin studies have provided evidence for a strong genetic component in individual gaze (Constantino et al., 2017; Kennedy, D'Onofrio, Quinn, Bölte, Lichtenstein, & Falck-Ytter, 2017).

Most relevant to the current study, observers freely viewing hundreds of complex scenes showed large individual differences in the number of fixated objects and in fixation tendencies toward objects from six semantic categories (de Haas, Iakovidis, Schwarzkopf, & Gegenfurtner, 2019). These differences were consistent across images and time and extended to first fixations after image onset, suggesting a bottom–up component.

Such semantic salience biases may be useful in the study of neurobiological mechanisms of attentional gaze control (de Haas et al., 2019) and form a crucial baseline for evaluating the diagnostic potential of gaze behavior for neurodevelomental and clinical conditions. In fact, research investigating eye movements in autism spectrum disorder (ASD) has demonstrated reduced social visual engagement in infants and adults with ASD compared to healthy controls (Constantino et al., 2017; Jones & Klin, 2013; Wang, Jiang, Duchesne, Laugeson, Kennedy, Adolphs, & Zhao, 2015). Others have found evidence for abnormal eye movements in patients with major depression (Armstrong & Olatunji, 2012). Further, patients with schizophrenia show a reduced number and spatial dispersion of fixations (Benson, Beedie, Shephard, Giegling, Rujescu, & St. Clair, 2012). However, the free viewing paradigm in de Haas et al. (2019) used the full stimulus set of the Object and Semantic Images and Eye-tracking (OSIE) dataset, comprised of 700 images (Xu et al., 2014). For practical purposes, it would be desirable to estimate individual gaze biases with a more economical test.

The present study had two main objectives. The first was to replicate the findings of stable individual gaze differences along semantic dimensions found in de Haas et al. (2019). Therefore, we tested whether the proportion of cumulative fixation times for objects of the categories *Text* and *Faces*, objects with implied *Motion*, objects with a characteristic *Taste*, or *Touched* objects vary reliably between observers. Further, we tested whether the proportion of first fixations varies reliably for objects of the categories *Text*, *Faces*, and *Touched*. We chose and preregistered these object dimensions, because they yielded large and consistent individual gaze differences (*r* > 0.6) in the original study by de Haas et al. (2019). Note, we combined neutral and emotional *Faces*, given the high covariance in fixation tendencies toward both. Moreover, we tried to replicate the findings of stable individual differences in visual exploration, as indicated by the number of objects an observer fixated. Second, we tested whether a smaller stimulus set can reliably estimate these differences. For this purpose, we selected subsets of OSIE images (OSIE_40_, OSIE_100_, and OSIE_200_), which a search algorithm predicted would yield high congruence with individual differences observed for the full set.

Two eye-tracking sessions were completed by 103 participants on separate days; one day they free-viewed the full OSIE set (700 images), and another day they free-viewed the smaller subsets. We then computed the correlation among individual fixation tendencies between the full and smaller sets to determine the minimum set size required for reliable estimates. Additionally, we repeated these analyses for gaze data truncated to the first 1 and 2 seconds of each trial (from a total of 3 seconds). Results showed that the most prominent individual fixation biases could be estimated reliably from just 40 images shown for 2 seconds each.

## Methods

### Subjects

A total of 103 healthy participants with normal or corrected-to-normal vision were recruited at Leibniz Institute of Psychology Information (ZPID) using their PsychLab offline service (*M_age_* *=* 25.17; *SD* = 5.50; seven left-handed; 72 females). The sample size was based on an a priori power analysis (de Haas, 2019). All participants took part in two sessions, with the second appointment following the first after an average of 16 days. The study was approved by the local ethics committee, and all participants gave written informed consent before participating.

### Apparatus

Stimuli were displayed on a BenQ XL2430T monitor (BenQ Corporation, Taipei, Taiwan) using Psychtoolbox 3.0.12 (Kleiner, Brainard, Pelli, Ingling, Murray, & Broussard, 2007; Pelli, 1997) in MATLAB R2019a (MathWorks, Natick, MA). Participants viewed the stimuli at a resolution of 1920 × 1080 pixels and at 29.0 × 22.2 degrees visual angle. Eye gaze from the left eye was measured using a desktop-mounted EyeLink 1000 Plus eye tracker (SR Research, Ottawa, Canada) at a frequency of 1 kHz.

### Stimuli and procedure

Participants completed free-viewing tasks that included a set of 700 images (day 1) and 200 images (day 2) with natural everyday scenes, each of which contained multiple objects. Semantic metadata for the full stimulus set consisted of binary pixel masks for 5551 objects and corresponding labels for 12 semantic dimensions as provided in the OSIE dataset (Xu et al., 2014). The provided labels were modified in order to minimize overlap between them, as in de Haas et al. (2019). Specifically, the *Smell* label was removed from all objects with the *Text* label, the *Operable* and *Gazed* labels were removed from all objects with the *Touched* label, and the *Watchable* label was removed from all objects with the *Text* label.

The design and procedure for both testing days were adopted from de Haas et al. (2019). After calibration, participants freely viewed seven blocks of 100 images each while sitting at a distance of ∼64 cm from the computer screen. Each trial started with the presentation of a central fixation disk followed by a 3-second presentation of the image that was initiated by a button press by the participant. All images appeared in the same order across participants. During the second appointment, this procedure was repeated with only two blocks (200 images).

The order of images presented during the first two blocks of the first day was determined a priori, based on the dataset by de Haas et al. (2019). An iterative search algorithm aimed at selecting images in an order that maximized the predictive validity for the full set at each step (i.e., for each number of images between 10 and 200, incrementing by one image). The order of images on the second day was pseudo-randomized relative to the first two blocks of the first day to minimize possible effects of order expectation. The order of images was randomized separately for images 1 to 40, 41 to 100, and 101 to 200 of the first day, but this division had no further relevance for the analyses performed here.

### Analysis

All statistical analyses were performed in MATLAB R2019a (MathWorks). Data processing closely followed the procedures in de Haas et al. (2019) and was preregistered (de Haas, 2019). To limit our dataset to object-directed fixations after stimulus onset, we excluded central onset fixations (onset time < 100 ms), fixations with duration below 100 ms (following standard settings from the eye tracker manufacturer), and fixations that could not be assigned a label. Fixations that fell on or within a distance of ∼0.5 degrees visual angle from a labeled object were assigned the corresponding label. To assess individual fixation tendencies, for each observer we computed the proportion of first fixations (after image onset) and of cumulative dwell time falling onto objects of a given label.

### Consistency and retest correlations

We used the data from the first testing day to replicate the study by de Haas et al. (2019), testing the split-half consistency of individual fixation proportions for each semantic dimension. This was repeated across 1000 random splits of images. We calculated two-sided *p* values for the median correlations across these splits, which were Bonferroni adjusted for the number of tested dimensions: *Faces*, *Text*, *Touched*, *Taste*, and *Motion* for cumulative dwell times and *Faces*, *Text*, and *Touched* for the proportion of first fixations, as preregistered (de Haas et al., 2019). Finally, we calculated the minimum/maximum ratios of individual fixations between observers and for each dimension. This was done by dividing the minimum percent cumulative dwell time and percent first fixation for a given dimension by the corresponding maximum across all observers.

To test the validity of estimates from smaller stimulus sets, we calculated fixation proportions as above, but for 190 subsets (from 10 to 200 images in steps of one). Next, we determined the correlation of individual differences for a given dimension and subset from day 2 with those observed for the full set on day 1. Note that only the preregistered set sizes of 40, 100, and 200 images contained identical images, because of the pseudo-randomized order between days (see above). We also probed the consistency of individual differences in the number of objects fixated, an indicator of the individual tendency for visual exploration. This part of the analyses was not preregistered but replicated de Haas et al. (2019).

Further, we explored the effects of shorter trial durations (also not preregistered). First, we probed the consistency of individual differences when truncating viewing data from day 1 to the first *n* milliseconds of each trial. For this analysis, we computed the proportion of cumulative dwell times for each semantic dimension considered and individual dwell times for different steps of truncation, from 100 ms to 3 seconds in steps of 100 ms. We then correlated these proportions with those observed for the full trial duration. Second, we computed split-half correlations between cumulative dwell time proportions across odd- and even-numbered images for each step of truncation. Third, we repeated all consistency analyses for smaller stimulus sets with gaze data trimmed to the first 1 and 2 seconds of each trial (data truncated for both day 1 and day 2).

To determine whether a subset of images (presented on day 2) yielded a valid estimate of individual differences along a given dimension, we tested its correlation with individual differences along the same dimension observed for the full set (on day 1). For this analysis, we applied a preregistered correlation threshold of *r* = 0.7. This criterion was very conservative, given the noise ceiling of full sets shown on separate days. Test–retest correlations for the full set ranged from *r* = 0.68 (*Motion*) to *r* = 0.84 (*Text*) in de Haas et al. (2019). We therefore additionally report the proportion of explainable variance explained by a given subset, relative to this estimate of the noise ceiling.

### Prevalence of image features across subsets

Moreover, to explore possible differences in the prevalence of image features that may lead to different consistency estimates across subsets, we additionally determined the relative and absolute number of occurrences for each semantic image feature for each subset.

### Data availability and preregistration

All anonymized data and code to reproduce the presented results, as well as the stimulus sets, are available at https://osf.io/ekvj4/. A preregistered study protocol is available at http://dx.doi.org/10.23668/psycharchives.2454.

## Results

**Figure 1.**
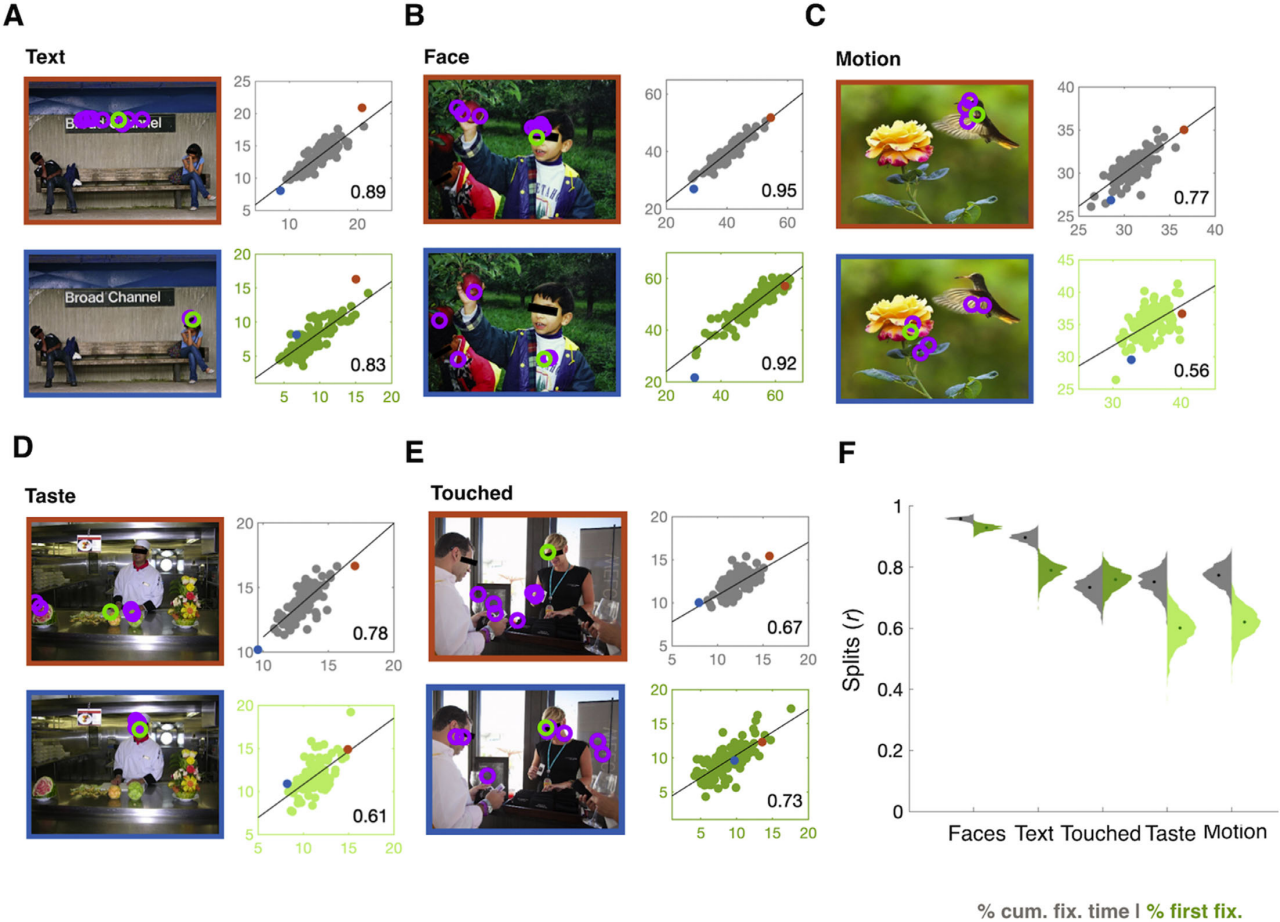
Consistency of fixation ratios. (A–E) Scatterplots show the split-half correlation between odd- versus even-numbered images for percent cumulative fixation time (gray) for the dimensions *Text*, *Faces*, *Motion*, *Taste*, and *Touched* and percent first fixations (green) for the dimensions *Text*, *Faces*, and *Touched*. These dimensions were preregistered and included based on the large and reliable individual differences along them (*r* > 0.6) reported by de Haas et al. (2019). Black inset numbers give the respective Pearson's correlation coefficient. For each dimension, one example is given showing the fixations from a participant with a strong tendency to fixate objects of the given dimension (orange frames) and one participant with a weaker tendency (blue frame). The green circles represent the first fixation after image onset, and purple circles represent any subsequent fixations. The orange and blue data points in the scatterplots correspond to the respective example participants and image frame color. Black bars in the images were added for display purposes only and were not seen during the free-viewing task. (F) Distribution of bootstrapped split-half correlations for each of the five included the semantic dimensions *Faces*, *Text*, *Touched*, *Taste*, and *Motion* (*x*-axis). The gray left-hand leaves show the distribution of split-half correlations for cumulative fixation time between image sets for 1000 random half-splits of images. The green right-hand leaves show the corresponding distributions for first fixations. Dots inside the distributions refer to the median correlation for each histogram and semantic dimension. Desaturated green scatterplots (A–D) and right-hand leaves (F) for the dimensions *Motion* and *Taste* are shown for completeness and were not preregistered (de Haas et al., 2019).

First, we replicated the large and consistent individual differences found by de Haas et al. (2019). Proportions of cumulative fixation time varied strongly among observers, with maximum/minimum ratios ranging from 1.37 (*Motion*) to 2.47 (*Text*). These differences were highly reliable across image splits for each semantic dimension (from *r* = 0.74, *p* < 0.001 for *Touched* to *r* = 0.96, *p* < 0.001 for *Faces*). Similar results were observed for the proportion of first fixations, with maximum/minimum ratios ranging from 2.40 for *Faces* to 3.47 for *Touched* and consistency ranging from *r* = 0.74, *p* < 0.001 for *Touched* to *r* = 0.93, *p* < 0.001 for *Faces* (Figure 1). Similarly, the number of distinct objects fixated consistently varied across observers, with a maximum/minimum ratio of 1.95 (*r* = 0.97, *p* < 0.001). Table 1 provides an overview of the results and gives the ranges of fixation proportions across observers.

**Table 1.** Median consistency and corresponding range of individual differences in fixation toward objects of five semantic dimensions. The left-hand side of the table shows results referring to individual differences in the proportion of cumulative dwell time toward objects of the five semantic attributes included in the study; the right-hand side displays results based on the proportion of first fixations landing on an object with a given attribute after image onset. Pearson correlations (*r*) indicate the median split-half correlation of individual differences measured over 1000 random image splits, and the corresponding *p* values are Bonferroni adjusted for the five semantic attributes. The range across observers is presented in percent cumulative fixation time and percent first fixation and the maximum/minimum ratio (max/min) indicates the maximum relative difference in individual gaze difference between observers.

	Cumulative fixation time				First fixations			
	*r*	*P*	Range (%)	Max/min	*r*	*P*	Range (%)	Max/min
*Faces*	0.96	<0.001	28–53	1.90	0.93	<0.001	26–63	2.40
*Text*	0.90	<0.001	8–21	2.50	0.79	<0.001	4–16	3.54
*Touched*	0.74	<0.001	9–16	1.73	0.76	<0.001	5–17	3.47
*Taste*	0.75	<0.001	10–17	1.71	0.61	<0.001	8–17	2.09
*Motion*	0.78	<0.001	26–36	1.37	0.62	<0.001	28–40	1.42

### Estimating individual differences with a smaller stimulus set

**Figure 2.**
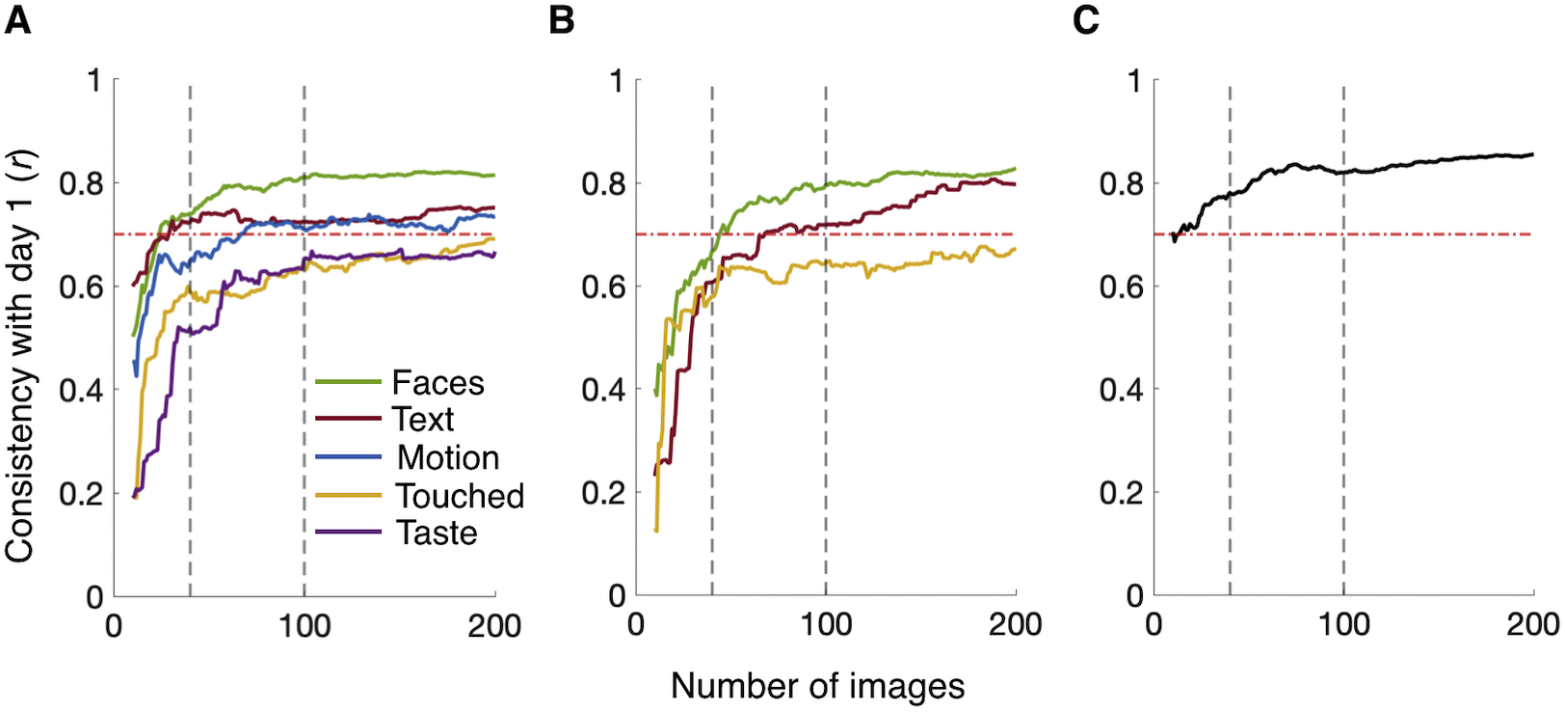
Consistency of fixation ratios based on smaller stimulus sets. Line plots depicting Pearson consistency correlations of individual differences in percent cumulative fixation time (A), percent first fixations (B), and visual exploration (number of distinct objects fixated) (C) between the full image set (day 1) and a given subset (10 to 200 images from day 2; *x*-axis). Dashed gray lines mark stimulus sets of 40 and 100 images. Dashed red lines indicate the consistency threshold of *r* = 0.7. Colors in (A) and (B) indicate semantic dimensions as shown in the inset.

Next, we probed whether these differences can be tested with a smaller set of images. Figure 2 shows the consistency (Pearson's *r*) of estimates for smaller stimulus sets from day 2 compared with those for the full dataset from day 1 as a function of set size, both for cumulative dwell time (Figure 2A) and first fixations (Figure 2B). Figure 2C shows the same analysis for visual exploration. For cumulative dwell times, a stimulus set of just 40 images yielded reliable estimates (*r* > 0.7, *p* < 0.001 with the full set) for individual differences along the dimensions *Text* and *Faces*. We found that 100 and 200 images reliably captured *Text*, *Faces*, and *Motion*, with the dimensions *Taste* and *Touched* being just below this threshold for the set of 200 images (*r* = 0.67, *p* < 0.001 for *Taste* and *r* = 0.69, *p* < 0.001 for *Touched*). For first fixations, a set of 100 images captured reliable inter-individual differences (*r* > 0.7, *p* < 0.001 with the full set) for *Text* and *Faces*. *Touched* came close to this threshold (*r* = 0.65, *p* < 0.001). Individual differences in visual exploration could reliably be estimated with a set size of 40 images (*r* = 0.78, *p* < 0.001).

### Estimating individual differences with shorter trial durations

**Figure 3.**
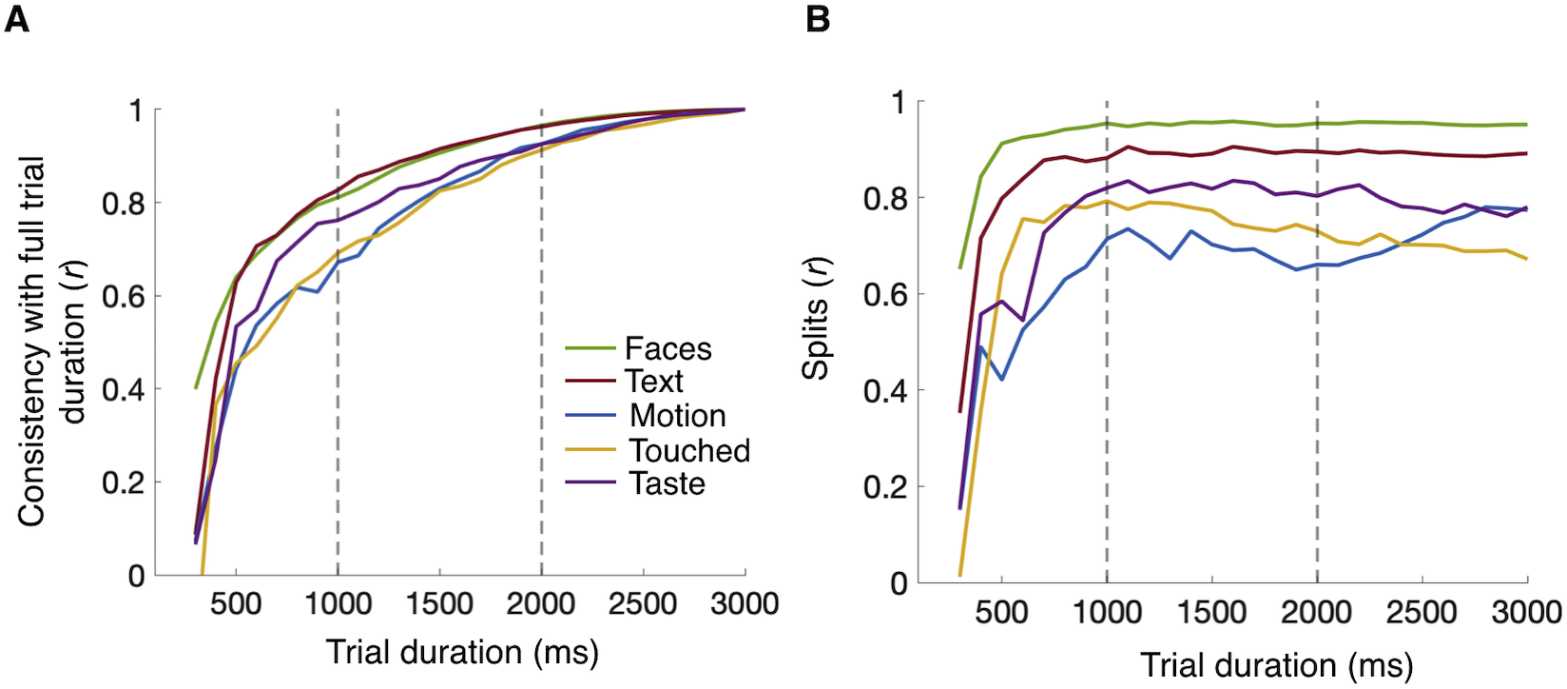
Consistency of estimates for truncated trial durations. (A) Line plots show the correlation of percent cumulative fixation time observed for the full trial duration with the corresponding values for different steps of truncation (starting from 1000 ms to 3000 ms in steps of 100 ms as shown on the *x*-axis; all data from day 1). (B) Line plot on the right-hand side presents split-half correlations across odd and even images for different steps of truncation. Dashed gray lines mark free viewing durations of 1000 ms and 2000 ms. Semantic dimensions are indicated by color as shown in the insets.

We further explored the effects of (virtually) shortened trial durations by trimming the data from each trial. Figure 3A considers estimated individual differences in cumulative dwell time from day 1 and shows the consistency correlation between these estimates based on the whole trial duration of 3 seconds versus the same estimates for trimmed durations. Figure 3B shows split-half reliabilities of estimates between odd and even images as a function of trimmed trial times. Across all tested dimensions, estimates based on the first 2 seconds of trials are highly consistent with those for the full trial duration (all *r* > 0.91, *p* < 0.001) and yield split-half reliabilities approaching or exceeding those seen for 3 seconds (ranging from *r* = 0.66, *p* < 0.001 for *Motion* to *r* = 0.95, *p* < 0.001 for *Faces*)*.*

### Combining small stimulus sets with shorter trial durations

**Figure 4.**
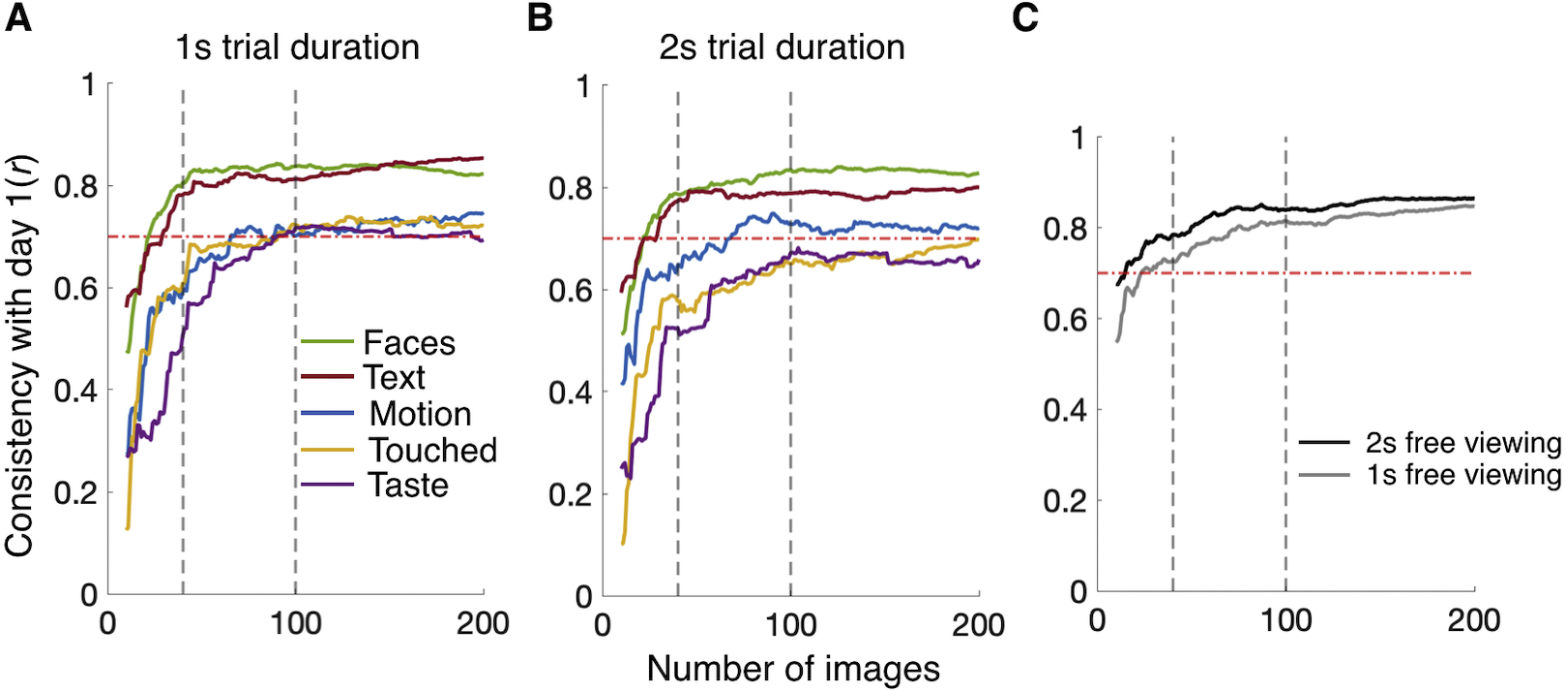
Consistency of estimates based on smaller stimulus sets for shorter trial durations. Line plots show the Pearson consistency of estimates for the full stimulus set (day 1) with smaller subsets from day 2. (A, B) Consistency correlations for estimated proportions of cumulative viewing times for trial durations trimmed to 1 second (A) and 2 seconds (B); semantic dimensions are indicated by color as shown in the inset. (C) Consistency correlations for the individual tendency for visual exploration (number of distinct objects fixated) for 1 second and 2 seconds as indicated by the inset. Dashed gray lines mark stimulus sets of 40 and 100 images. Dashed red lines indicate the desired consistency threshold of *r* = 0.70.

Next, we considered the effects of trimmed trial durations on smaller stimulus sets. Figure 3C and Figures 4A and 4B show the number of images necessary to estimate reliable individual differences (*r* ≥ 0.7) in cumulative dwell time as a function of (trimmed) trial duration. *Faces* and *Text* reached this criterion with fewer than 40 images for all trial durations. *Motion* could reliably be estimated with fewer than 100 images when using at least the first second of trial data. Interestingly, *Touched* and *Taste* crossed the threshold with fewer than 100 images for the first second of trial duration (Figure 4A) but required more than 100 (*Touched*) or even 200 (*Taste*) images for trial durations of 2 seconds and 3 seconds. Figure 4C shows the consistency of individual visual exploration (numbers of objects fixated) with day 1 as a function of trial duration and considered set size from day 2. At 40 images, the data are above the consistency threshold for both 1-second and 2-second trial durations. Supplementary Table S1 provides an overview of results and compares consistency correlations to the proportion of explainable variance (as indicated by test–retest reliabilities for the full set reported by de Haas et al., 2019).

### Prevalence of image features

**Figure 5.**
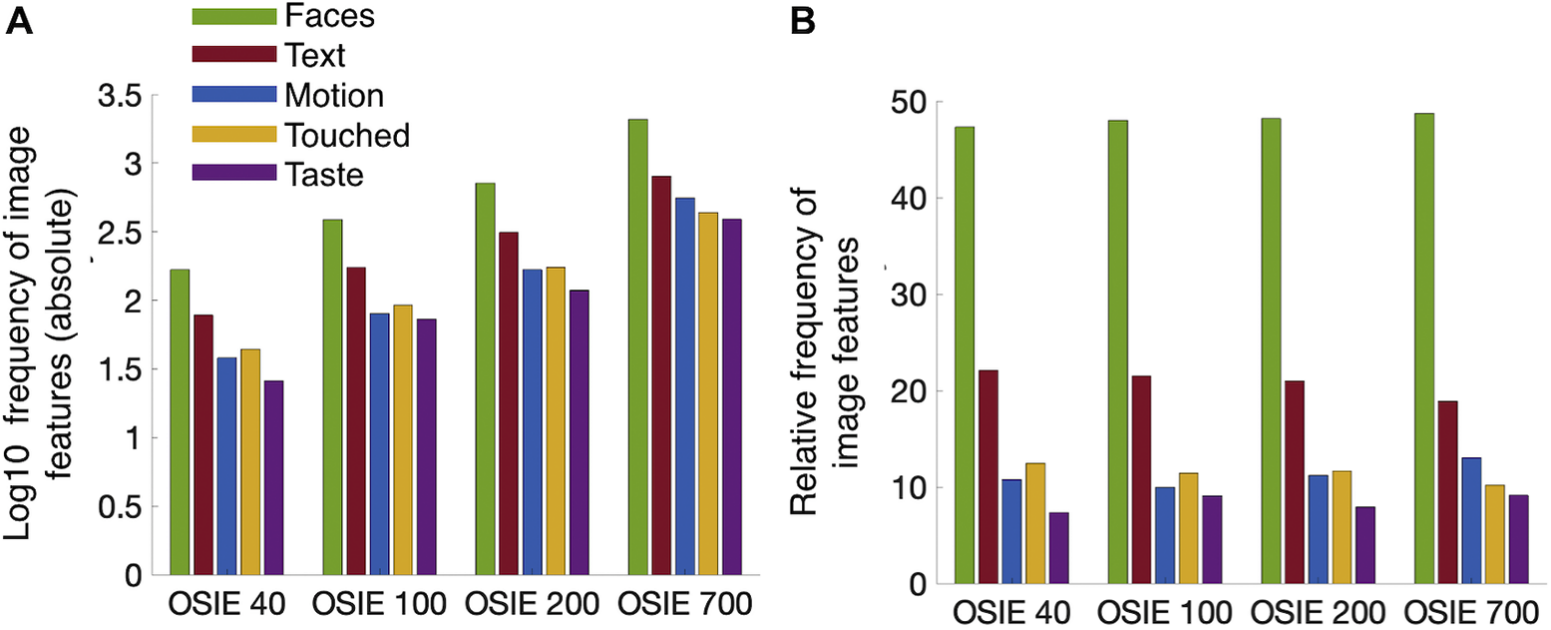
Prevalence of image features along all semantic dimensions and across image sets. (A) Bar plot describing the absolute frequency (log10 scale) of image features for a given semantic dimension and across image sets. (B) Bar plot displaying the relative frequency in percent for image features of a given dimension and image set. Semantic dimensions are indicated by color as shown in the inset.

In a final step we determined the prevalence of image features for the image sets OSIE_40_, OSIE_100_, OSIE_200_, and OSIE_700_. Figure 5 shows the (log10) absolute frequency (Figure 5A) and the relative frequency (Figure 5B) of these features across stimulus sets. Results show that the relative distribution of features was stable across image sets (Figure 5B). The proportion of *Faces* was most similarly distributed across image sets and ranged from 47.31 (OSIE_40_) to 48.74 (OSIE_700_). The proportion of *Text* was least stable and ranged from 18.88 (OSIE_700_) to 22.1 (OSIE_40_). However, the absolute number of all feature occurrences (unsurprisingly) was heavily reduced for the shorter sets (Figure 5A).

## Discussion

The results of the present study allow two conclusions. First, they corroborate the finding of reliable individual differences in gaze behavior reported by de Haas et al. (2019). Individual differences in visual exploration and systematic fixation biases toward a set of semantic features were replicated for both dwell time and first fixations. Second, the present results show how to test these differences efficiently.

Based on our results, we propose the OSIE_40_ subset with a presentation time of 2 seconds per image as an attractive option for estimating individual gaze behavior efficiently. When using the re–test reliabilities for the full image set reported by de Haas et al. (2019) as an estimate of the noise ceiling, this test captures more than 80% of the explainable variance in visual exploration and dwell time for three out of five dimensions (*Face*, *Text*, and *Motion*) and 69% of explainable dwell-time variance for *Touched*. The remaining fifth dimension of *Taste* was not well captured by any of the short tests, which perhaps is unsurprising given its relatively low internal consistency, as reported by de Haas et al. (2019) and replicated here.

Researchers interested in individual differences in first fixations can achieve acceptable results using the OSIE_100_ but can expect considerable gains from using the full stimulus set. All subsets of stimuli can be retrieved from Supplementary Table S2 and downloaded with the online data and code at https://osf.io/ekvj4/.

Exploratory analyses showed that the relative prevalence of image features was similarly distributed across dimensions for the different image subsets (Figure 5B). Nevertheless, it is possible that the lower absolute number of features in the image subsets caused floor effects for consistency estimates of less salient dimensions (i.e., *Touched* and *Taste*) (de Haas et al., 2019; Xu et al., 2014), which also appear less frequently in the images. The ability to estimate individual differences in fixation tendencies along a given dimension depends on sufficient data. If the number of relevant fixations in the dataset is too low, it will lack the necessary granularity to capture smaller but relevant inter-individual differences. It seems likely that the relatively low saliency for *Taste* and *Touched* (de Haas et al., 2019; Xu et al., 2014) in the smaller image sets is compounded by a low absolute number of occurrences of these features, yielding such floor effects.

Individual differences in gaze behavior are gaining interest in vision and computer science (Li & Chen, 2018; Yu & Clark, 2017), as well as clinical psychology (Armstrong & Olatunji, 2012). Especially in developmental studies and clinical settings, free-viewing experiments using natural images are ideal because of their minimum task requirements. Fixation measurements also come with minimal technical requirements and could soon be bedside compatible. At the same time, applied settings and clinical research often come with severe time limits and prohibit the use of extensive testing batteries. We hope our current results contribute to closing this gap and will provide a useful standard for researchers aiming to explore individual fixation tendencies in a time-efficient manner.

The explorative analyses of truncating trial durations suggested that individual differences can reliably be estimated based on trials of just 1 or 2 seconds rather than 3 seconds. Notably, we observed slightly higher validity correlations for 1 second versus 2 and 3 seconds for the dimensions *Touched* and *Taste* (Figures 3A, 3B). Although these consistency differences between truncated datasets were small, they were constant across subsets. A possible reason for this is that individual fixations tendencies toward *Touched* objects or objects with *Taste* are only stable insofar as they are guided by bottom–up processes and have a higher degree of intra-subject variability as soon as later, top–down processes come into play. For example, earlier fixations toward objects with *Taste* may be driven by relatively stable individual levels of saliency for low-level features, such as the high color contrast of these objects. Later, top–down-directed fixations may in turn more heavily depend on the variable states of the observer, such as hunger or satiety.

Further, these analyses were based on trimming the viewing data rather than the actual viewing duration during the experiment. We cannot exclude the possibility that shortening trial times during testing would have different and maybe unexpected consequences due to the altered pace of the experiment. Pilot data from our lab suggest that experiments with a 2-second viewing duration yield individual differences with excellent internal consistency; however, this comes with an intercept shift across the given semantic dimensions. Future research could aim for an in-depth comparison of 2-second and 3-second trial durations in the same sample.

In the present study fixation differences were assessed with a free-viewing task. Past research has demonstrated that low-level properties of gaze behavior and the low-level features of fixated regions vary between tasks and can also interact with individual differences (Kardan, Berman, Yourganov, Schmidt, & Henderson, 2015; Kardan, Henderson, Yourganov, & Bergmann, 2016). However, these studies did not consider individual differences in semantic saliency. Future research should test whether saliency along these dimensions is modulated by task and in particular whether such modulations are limited to intercept shifts on the group level or interact with individual differences.

In sum, we have replicated consistent individual differences in gaze behavior and documented that they can be estimated using less than 2 minutes of free-viewing data.

## Supplementary Material

Supplement 1
