# Supplementary material for: OSIEshort: A small stimulus set can reliably estimate individual differences in semantic salience
Source: J Vis. 2020 Sep 18;20(9):13. doi: 10.1167/jov.20.9.13 (PMC7509791; doi:10.1167/jov.20.9.13)
Supplement: Supplement 1 [file jovi-20-9-13_s001.docx]

**Supplementary materials**

**Table S1**

*Consistency correlations for small stimulus sets and truncated trial durations.* Rows show consistency correlations for each semantic dimension and visual exploration for the OSIE_40_, _100_ and _200_ sets. Columns show Pearson consistency correlations (r) between the estimated proportion of individual dwell time (or first fixations rightmost columns) on the respective objects for the full set seen on day 1 and the respective subset on day 2. Dwell time estimates from day 2 were either based on the full trial duration (3s) or trials truncated to 1 or 2s, as indicated by the column headings. % expl. indicates which proportion of explainable variance a given consistency correlation corresponds to. This was estimated based on the test-retest reliabilities for the full stimulus set (700 images) reported by de Haas et al. (2019), which served as an independent estimate of the noise-ceiling.

|  | Cumulative fixation time | | | | |  | First fixations | |
| --- | --- | --- | --- | --- | --- | --- | --- | --- |
|  | Trial dur. (1s) | | **Trial dur. (2s)** | | Trial dur. (3s) | |  | |
|  | *r* | % exp. | ***r*** | **% exp.** | *r* | % exp. | *r* | % exp. |
| OSIE_40_ |  |  |  |  |  |  |  |  |
| Faces | .81 | 103 | **.79** | **98** | .74 | 86 | .67 | 61 |
| Text | .78 | 86 | **.77** | **84** | .72 | 73 | .60 | 46 |
| Motion | .59 | 75 | **.65** | **91** | .65 | 91 | .51 | 43 |
| Touched | .61 | 76 | **.58** | **69** | .59 | 71 | .58 | 55 |
| Taste | .51 | 43 | **.53** | **46** | .51 | 43 | .38 | 32 |
| Vis. exploration | .73 | 83 | **.79** | **98** | .78 | 95 | - | - |
| OSIE_100_ |  |  |  |  |  |  |  |  |
| Faces | .84 | 110 | .83 | 108 | .81 | 103 | **.80** | **86** |
| Text | .81 | 93 | .79 | 88 | .72 | 73 | **.72** | **65** |
| Motion | .70 | 106 | .73 | 115 | .71 | 109 | **.54** | **48** |
| Touched | .72 | 106 | .65 | 86 | .63 | 81 | **.65** | **69** |
| Taste | .72 | 85 | .67 | 74 | .65 | 69 | **.57** | **72** |
| Vis. exploration | .82 | 105 | .84 | 110 | .82 | 105 | - | - |
| OSIE_200_ |  |  |  |  |  |  |  |  |
| Faces | .82 | 105 | .83 | 108 | .82 | 105 | .83 | 93 |
| Text | .85 | 102 | .80 | 91 | .75 | 80 | .81 | 79 |
| Motion | .75 | 122 | .72 | 112 | .73 | 115 | .65 | 69 |
| Touched | .72 | 106 | .70 | 100 | .69 | 97 | .67 | 74 |
| Taste | .69 | 78 | .66 | 72 | .67 | 74 | .63 | 88 |
| Vis. exploration | .85 | 113 | .86 | 116 | .85 | 113 | - | - |

**Table S2**

*Images included in the three tested stimulus subsets OSIE_40,_ OSIE_100_ and OSIE_200_*

| Image number | | |
| --- | --- | --- |
| OSIE_40_ | OSIE_100_ | OSIE_200_ |
| 1608 | 1608 | 1608 |
| 1226 | 1226 | 1226 |
| 1233 | 1233 | 1233 |
| 1335 | 1335 | 1335 |
| 1472 | 1472 | 1472 |
| 1391 | 1391 | 1391 |
| 1479 | 1479 | 1479 |
| 1277 | 1277 | 1277 |
| 1590 | 1590 | 1590 |
| 1118 | 1118 | 1118 |
| 1296 | 1296 | 1296 |
| 1094 | 1094 | 1094 |
| 1178 | 1178 | 1178 |
| 1460 | 1460 | 1460 |
| 1101 | 1101 | 1101 |
| 1620 | 1620 | 1620 |
| 1576 | 1576 | 1576 |
| 1287 | 1287 | 1287 |
| 1265 | 1265 | 1265 |
| 1566 | 1566 | 1566 |
| 1573 | 1573 | 1573 |
| 1420 | 1420 | 1420 |
| 1188 | 1188 | 1188 |
| 1227 | 1227 | 1227 |
| 1433 | 1433 | 1433 |
| 1112 | 1112 | 1112 |
| 1614 | 1614 | 1614 |
| 1001 | 1001 | 1001 |
| 1606 | 1606 | 1606 |
| 1643 | 1643 | 1643 |
| 1567 | 1567 | 1567 |
| 1522 | 1522 | 1522 |
| 1526 | 1526 | 1526 |
| 1480 | 1480 | 1480 |
| 1543 | 1543 | 1543 |
| 1544 | 1544 | 1544 |
| 1491 | 1491 | 1491 |
| 1006 | 1006 | 1006 |
| 1132 | 1132 | 1132 |
| 1481 | 1481 | 1481 |
|  | 1247 | 1247 |
|  | 1586 | 1586 |
|  | 1187 | 1187 |
|  | 1550 | 1550 |
|  | 1441 | 1441 |
|  | 1395 | 1395 |
|  | 1468 | 1468 |
|  | 1254 | 1254 |
|  | 1182 | 1182 |
|  | 1579 | 1579 |
|  | 1246 | 1246 |
|  | 1009 | 1009 |
|  | 1286 | 1286 |
|  | 1527 | 1527 |
|  | 1035 | 1035 |
|  | 1138 | 1138 |
|  | 1148 | 1148 |
|  | 1425 | 1425 |
|  | 1096 | 1096 |
|  | 1694 | 1694 |
|  | 1637 | 1637 |
|  | 1268 | 1268 |
|  | 1663 | 1663 |
|  | 1456 | 1456 |
|  | 1162 | 1162 |
|  | 1301 | 1301 |
|  | 1349 | 1349 |
|  | 1621 | 1621 |
|  | 1628 | 1628 |
|  | 1011 | 1011 |
|  | 1416 | 1416 |
|  | 1022 | 1022 |
|  | 1484 | 1484 |
|  | 1128 | 1128 |
|  | 1474 | 1474 |
|  | 1229 | 1229 |
|  | 1580 | 1580 |
|  | 1023 | 1023 |
|  | 1353 | 1353 |
|  | 1565 | 1565 |
|  | 1308 | 1308 |
|  | 1103 | 1103 |
|  | 1169 | 1169 |
|  | 1015 | 1015 |
|  | 1578 | 1578 |
|  | 1539 | 1539 |
|  | 1452 | 1452 |
|  | 1236 | 1236 |
|  | 1018 | 1018 |
|  | 1531 | 1531 |
|  | 1583 | 1583 |
|  | 1327 | 1327 |
|  | 1209 | 1209 |
|  | 1197 | 1197 |
|  | 1248 | 1248 |
|  | 1044 | 1044 |
|  | 1385 | 1385 |
|  | 1602 | 1602 |
|  | 1465 | 1465 |
|  | 1352 | 1352 |
|  |  | 1208 |
|  |  | 1570 |
|  |  | 1386 |
|  |  | 1431 |
|  |  | 1325 |
|  |  | 1204 |
|  |  | 1547 |
|  |  | 1382 |
|  |  | 1445 |
|  |  | 1106 |
|  |  | 1568 |
|  |  | 1309 |
|  |  | 1406 |
|  |  | 1670 |
|  |  | 1288 |
|  |  | 1478 |
|  |  | 1366 |
|  |  | 1077 |
|  |  | 1683 |
|  |  | 1363 |
|  |  | 1303 |
|  |  | 1401 |
|  |  | 1657 |
|  |  | 1358 |
|  |  | 1508 |
|  |  | 1430 |
|  |  | 1540 |
|  |  | 1377 |
|  |  | 1372 |
|  |  | 1210 |
|  |  | 1111 |
|  |  | 1113 |
|  |  | 1562 |
|  |  | 1455 |
|  |  | 1059 |
|  |  | 1279 |
|  |  | 1130 |
|  |  | 1109 |
|  |  | 1512 |
|  |  | 1646 |
|  |  | 1313 |
|  |  | 1356 |
|  |  | 1273 |
|  |  | 1461 |
|  |  | 1556 |
|  |  | 1002 |
|  |  | 1675 |
|  |  | 1075 |
|  |  | 1680 |
|  |  | 1381 |
|  |  | 1088 |
|  |  | 1376 |
|  |  | 1393 |
|  |  | 1219 |
|  |  | 1462 |
|  |  | 1206 |
|  |  | 1292 |
|  |  | 1257 |
|  |  | 1284 |
|  |  | 1241 |
|  |  | 1412 |
|  |  | 1440 |
|  |  | 1156 |
|  |  | 1196 |
|  |  | 1518 |
|  |  | 1215 |
|  |  | 1624 |
|  |  | 1592 |
|  |  | 1351 |
|  |  | 1302 |
|  |  | 1281 |
|  |  | 1150 |
|  |  | 1609 |
|  |  | 1650 |
|  |  | 1483 |
|  |  | 1528 |
|  |  | 1551 |
|  |  | 1546 |
|  |  | 1146 |
|  |  | 1244 |
|  |  | 1216 |
|  |  | 1272 |
|  |  | 1371 |
|  |  | 1079 |
|  |  | 1147 |
|  |  | 1258 |
|  |  | 1548 |
|  |  | 1131 |
|  |  | 1183 |
|  |  | 1238 |
|  |  | 1700 |
|  |  | 1192 |
|  |  | 1555 |
|  |  | 1201 |
|  |  | 1501 |
|  |  | 1593 |
|  |  | 1502 |
|  |  | 1119 |
|  |  | 1340 |
|  |  | 1610 |
